# Supplementary material for: Determinants of women’s empowerment in Nepal
Source: PLoS One. 2024 Sep 11;19(9):e0310266. doi: 10.1371/journal.pone.0310266 (PMC11389927; doi:10.1371/journal.pone.0310266)
Supplement: Attachment — Submitted filename: Response to Reviewers.docx [file pone.0310266.s001.docx]

Dear Editor and Referee,

We would like to thank you for the feedback and the comments that helped to improve the paper!

We are submitting a revised manuscript along with point-by-point responses in the Table below.

We have made the requested revisions and believe that these changes have further improved the manuscript.

Sincerely,

The Authors

Editor

| Comment | Response |
| --- | --- |
| Although your paper is interesting, the reviewer had provided a variety of important concerns and helpful suggestions. Please respond to each comment of the reviewer carefully and thoroughly. In particular, please pay attention to those fundamental statistical issues raised by the reviewer. Moreover, please enrich the Discussion section. Please explain where you feel you cannot completely agree with reviewers’ suggestions, if any. | We agree with all the suggestions and incorporated them into the paper.  In particular, we addressed the fundamental statistical issues as explained below.  We have also substantially extended the discussion in the paper. |

Reviewer

| Comment | Response |
| --- | --- |
| − Please explain why you categorize the age variable. While there are intervention reasons (as alluded to in the discussion section), statistically information is lost when you categorize. I am not asking you to re-run your data with the continuous age variable but do provide your reader with a justification. | Our study uses a methodology similar to an earlier study based on a DHS survey in Mozambique (Castro, Lopes et al., 2021). Hence, for comparability of the results between Nepal and Mozambique we categorize age (and other) variables in the same way. We added an explanation on this account in the section “Independent variables”. |
| − PCA uses continuous variables, please explain how you used categorical variables. And to clarify, it is the variables in Table 1 that were used in the PCA to generate the empowerment index—correct? Please clarify. | Indeed, this is a very important point which needed clarification! We added the following clarification to text in the “Methodology” section:  “When applying PCA we followed Castro Lopes et al. (2021) with ordinal encoding for the empowerment indexes, coding them into a 3-point scale (i.e., values of -1, 0, 1) where the highest value was assigned to categories representing greater levels of empowerment. In particular, we used variables from Table 1 in the PCA to generate the empowerment indexes.” |
| − Reference Table 3 somewhere within the text. Are these results from the logistic regression models? | We would like to thank the reviewer for pointing out to our oversight – we have now introduce Table 3 in the manuscript right before discussing the results in this Table. |
| − The provincial breakdown in the bivariate analysis is very much appreciated. Please tell us a little bit about the geo-political structure of these provinces to help us contextualize your findings. | This is a very good point that we now address in the manuscript. In particular, we added the discussion in the beginning of the section “Results” which discusses geographic, political, economic and other differences that are further confirmed by our analyses in Table 1 and elsewhere. |
| − I was also expecting a little more critical analysis in the discussion section more so given the compelling regional comparisons presented in the results section. However, I find this section rather generic. For instance, what policies/programs/interventions stand to benefit from these results? What can health/social welfare practitioners glean from these results? | We have addressed this important concern by substantially extending the “Discussion” section so that welfare practitioners in Nepal can get important insights from our analyses. In particular, we added two paragraphs on appropriate policies and interventions that can be implemented in provinces that fall behind on a specific women empowerment index. |
| All the best! | Thank you for your helpful suggestions! |
